# Comparing the effect of virtual reality and transcranial direct current stimulation on fear of falling, balance, and walking ability in stroke: A randomized controlled trial protocol

**DOI:** 10.1016/j.mex.2026.104161

**Published:** 2026-09-11

**Authors:** Akram Divandari, Iraj Abdollahi, Mohammad Hassan Azarsa, Mohammad Sayyadnasiri, Neda Mostafaee

**Affiliations:** a Neuromusculoskeletal Rehabilitation Research Center, Department of Physiotherapy, University of Social Welfare and Rehabilitation Sciences, Tehran, Iran; b Clinical Research Development Center of Rofeideh Rehabilitation Hospital, University of Social Welfare and Rehabilitation Sciences, Tehran, Iran; c Iranian Center of Excellence in Physiotherapy, Rehabilitation Research Center, Department of Physiotherapy, School of Rehabilitation Sciences, Iran University of Medical Sciences, Tehran, Iran

**Keywords:** Stroke, Fear of falling, Balance, Walking ability, VR, TDCS

## Abstract

Fear of falling is a prevalent and disabling problem after stroke, contributing to reduced activity, impaired balance, and increased fall risk. Recent studies indicate that virtual reality (VR)–based interventions and transcranial direct current stimulation (tDCS) applied during exercise therapy may help reduce fear of falling in individuals with neurological disorders.

This single‑center, assessor‑blinded randomized controlled trial aims to compare the effects of VR‑based gait training with tDCS combined with gait training on fear of falling in patients with subacute stroke. Participants will be randomly allocated to either the VR or tDCS group, while all also receive conventional physiotherapy. Outcomes will be assessed at baseline and after ten treatment sessions by a blinded assessor. The primary outcome is fear of falling, measured using the Falls Efficacy Scale‑International (FES‑I). Secondary outcomes include balance, assessed by the Mini‑BESTest and Biodex Balance System, as well as walking ability, assessed by the 10‑Meter Walk Test. It is hypothesized that VR‑based gait training will result in a greater reduction in fear of falling than tDCS combined with gait training.

## Graphical abstract

**Figure:**
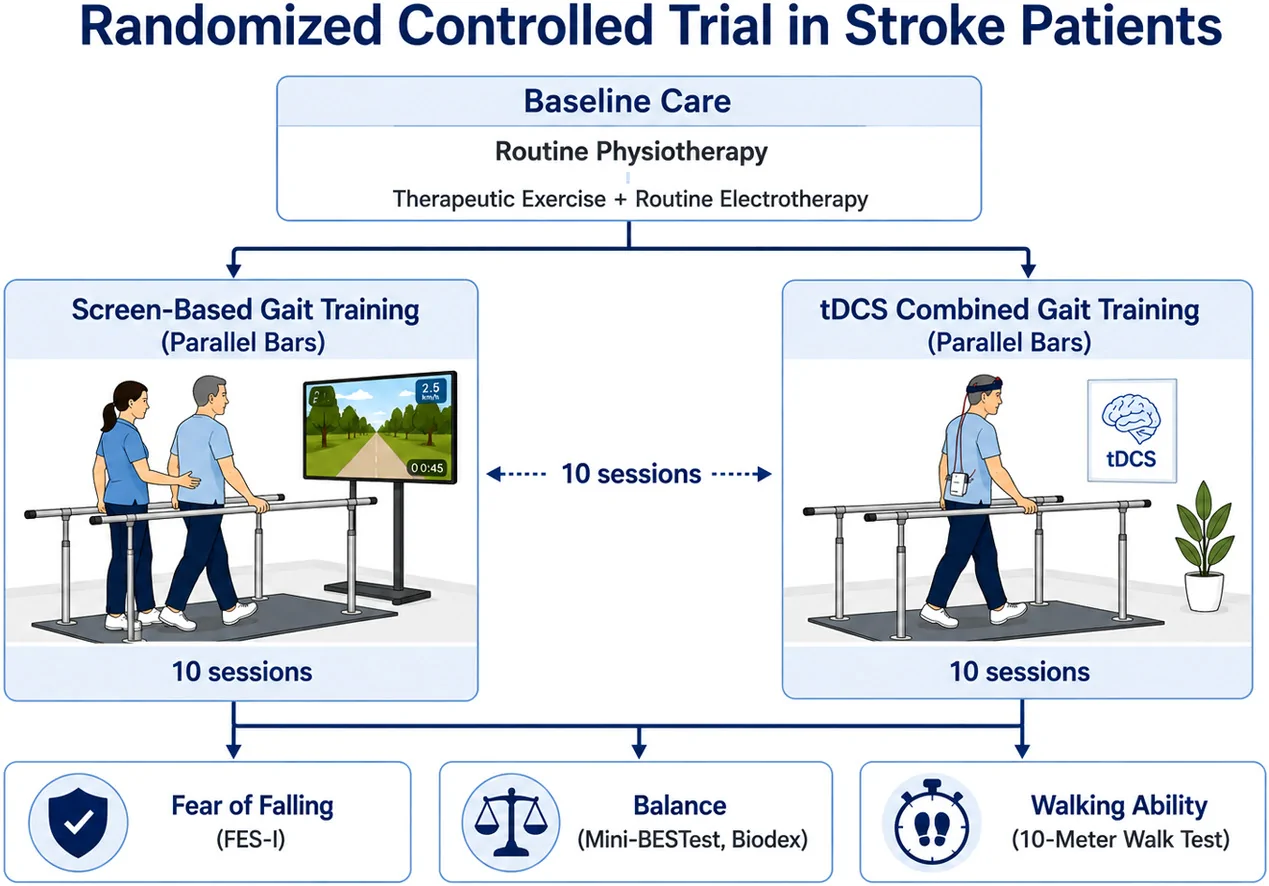

Specifications table

**Table:** 

**Subject area**	Neuroscience
**More specific subject area**	*Fear of falling*
**Name of your protocol**	*Comparing the effect of virtual reality and transcranial direct current stimulation on fear of falling, balance, and walking ability in patients with stroke*
**Reagents/tools**	• The Falls Efficacy Scale International questionnaire • The shortened version of the Balance Evaluation Systems Test • The Biodex Balance System • The 10-Meter Walk Test
**Experimental design**	*Stroke patients will be allocated to two intervention groups, Virtual Reality and transcranial Direct Current Stimulation combined with gait training . All participants will receive conventional physiotherapy also. Group allocation will be performed using block randomization stratified by sex and history of falls. The outcome measures include fear of falling, balance, and walking ability. These variables will be assessed at prior to the initiation of treatment and the day after the last session.*
**Trial registration**	*Registration No.: IRCT20251005067515N1*
**Ethics**	• The study will be conducted in accordance with the Declaration of Helsinki and approved by the Ethics Committee of the University of Social Welfare and Rehabilitation Sciences (IR.USWR.REC.1404.126). • Written informed consent will be obtained, and participants will be informed of study procedures, confidentiality, and their right to withdraw at any time.
**Value of the Protocol**	• To compare the effects of virtual reality versus tDCS during walking on fear of falling in stroke patients. • To evaluate the potential of technology‑assisted rehabilitation to enhance post‑stroke recovery. • To provide clinical evidence on the applicability and effectiveness of VR‑ and tDCS‑based interventions in routine stroke rehabilitation.

## Background

Stroke remains one of the most significant public health challenges, ranking as the second cause of death in older adults and fifth among younger populations. Annually, about 15 million people suffer a stroke, leading to millions of deaths and long-term disabilities [1].

Falling represents significant barrier to functional recovery and community reintegration in individuals who have sustained a stroke. Research findings on predictors of falls indicate that fear of falling is the third most important contributor to falls in stroke patients, following physical impairments in balance control and muscle strength during walking [2,3]. This fear can initiate a detrimental cycle: the ‘vicious cycle of falling’, where fear leads to reduced activity, which in turn impairs balance, increasing the likelihood of falls. These falls then intensify the initial fear, perpetuating a cycle that hinders effective post-stroke rehabilitation [4]. Consequently, Fear of falling is regarded as a psychological–cognitive factor rather than merely a consequence, underscoring the importance of early identification and timely intervention to disrupt this maladaptive cycle [5]. Recognizing the limitations of conventional gait training alone for achieving higher-level recovery in stroke patients, recent systematic reviews advocate for combined and innovative exercise approaches [6].

Among these, Virtual Reality (VR) technology allows for the creation of ecologically valid, yet controlled, environments that challenge postural control and gait parameters. VR platforms provide immediate, quantifiable, and personalized visual feedback regarding performance metrics such as step length variability, gait symmetry, and deviation from a target path[7]. Several studies have reported the beneficial effects of virtual reality on older adults, balance in patients with stroke, and reduction of fear of falling in patients with Parkinson’s disease and multiple sclerosis [[8], [9], [10], [11]]. This intensive, repetitive practice, coupled with extrinsic feedback, is hypothesized to drive neuroplastic changes facilitating improved motor learning and, consequently, reduced FoF^1^ and enhanced balance [12].

Another promising intervention is Transcranial Direct Current Stimulation (tDCS), a non-invasive technique that modulates cortical excitability using weak electrical currents. Targeting the dorsolateral prefrontal cortex (DLPFC) is relevant as this area is heavily implicated in cognitive control, executive function, planning, and the regulation of movement and fear/anxiety responses [13,14]. Several studies have shown that tDCS improves balance and gait parameters in older adults and individuals with stroke [[15], [16], [17]]. Moreover, tDCS is currently being used as a recognized guideline-based intervention for reducing fear and stress [18,19]. By employing anodal stimulation over the DLPFC during active motor tasks (walking), we aim to enhance cortical excitability in relevant motor networks, potentially augmenting the benefits derived from the concurrent physical training and promoting more robust motor relearning and confidence building [15,20].

This randomized controlled trial compares virtual reality and tDCS‑assisted walking. hypothesizing that VR, through real‑time multisensory stimulation and engagement of the mirror neuron system, produces greater effects than tDCS, which primarily targets central nervous system–mediated stress reduction and neuroplasticity.

## Description of protocol

### Study design

This study is a single-center, assessor- and analyzer-blinded, parallel-group randomized controlled trial comparing two high-intensity rehabilitation approaches: VR-based gait training and tDCS applied concurrently with parallel-bar gait training. The trial will be conducted at Rofeideh Hospital, affiliated with the University of Rehabilitation Sciences and Social Health, Tehran, Iran, where all intervention sessions and outcome assessments will take place (Table 1).

**Table 1:** Participant flow of enrolment, assessments and interventions.

	T0	T1	TT	T2
*Recruitment*	*			
*Interview (inclusion/exclusion)*	*			
*Assigning Consent form*		*		
*Randomization*		*		
*Questionnaires (HADS; MMSE; FES-I; Mini-BEST; 10MWT)*		*		*
*Assessment of balance (Biodex balance system)*		*		*
*VR, tDCS training intervention*			*	

T0, prior to study; T1, baseline (pre-intervention); TT, training time; T2, post-intervention test. HADS, Hospital Anxiety and Depression Scale; MMSE, Mini-Mental Scale Examination; FES-I, Falls Efficacy Scale International; Mini-BEST, shortened version of the BESTest, 10MWT; 10‑Meter Walk Test.

### Inclusion and exclusion criteria

32 stroke patients will be recruited for this study. inclusion criteria: Age between 40 and 70 years [21]. 2. Confirmed diagnosis of unilateral ischemic stroke in the subacute phase (3–6 months post-onset) [6] 3. Ability to walk independently for a minimum of 10 m, potentially with the assistance of a single standard walking aid [22]. 4. Hospital Anxiety and Depression Scale (HADS) score < 16 to ensure participants’ ability to cooperate and to exclude cases of severe depression [23]. 5. Cognitive screening score ≥ 21 on the Mini-Mental State Examination (MMSE) [24]. 6. Spasticity severity of the ankle dorsiflexor group, according to the Modified Ashworth Scale (MAS), on the injured side 2 or less [25]. Exclusion Criteria: 1. Significant orthopedic, vestibular, or cardiovascular conditions precluding safe gait training. 2. Presence of implanted metallic devices, pacemakers, deep brain stimulators. 3. Presence of major neurological comorbidities or psychiatric disorders. Participant recruitment will take place through the inpatient neurological rehabilitation wards of Rofeideh Hospital and affiliated units of URSSH. Throughout all treatment sessions, if the participant experiences severe fatigue, blurred vision, dizziness, or any other unexpected adverse sensation, the intervention will be immediately discontinued. The trial will also be stopped for any participant who shows sensitivity or intolerance to tDCS, expresses unwillingness to continue participation, or develops any of the exclusion criteria during the study period.

A blinded assessor will evaluate outcomes at baseline and after ten physiotherapy sessions, with assessments scheduled to avoid unblinding. The study follows the Declaration of Helsinki, written informed consent will be obtained, and participants’ demographic and clinical information will be recorded after randomization.

### Random allocation

participants will be randomly assigned in a 1:1 ratio to either the VR-based rehabilitation group or the tDCS group. Block randomization will be performed, stratified by sex and history of falling (presence or absence of previous falls). A computer-generated randomization sequence will be created by an independent statistician, and allocation concealment will be ensured using sealed, opaque envelopes.

### Interventions

Both groups will receive a standardized, supervised rehabilitation program consisting of 10 sessions during 1 month (one day in between). Each treatment session in both groups lasted 60 minutes and consisted of three parts: 20 minutes of functional electrical stimulation applied to the quadriceps and tibialis anterior muscles (10 minutes each, 35 Hz frequency, 200–400 µs pulse width, with current intensity producing a clear, painless contraction); 20 minutes of conventional therapeutic exercises—including strengthening of the tibialis anterior, quadricep, gluteal and hamstring to improve stance phase control and functional activities such as sit to stand, step up, push up [6]; and finally, 20 minutes of gait training using parallel bars under therapist supervision.

#### VR‑based gait training group

**Fig. 1:**
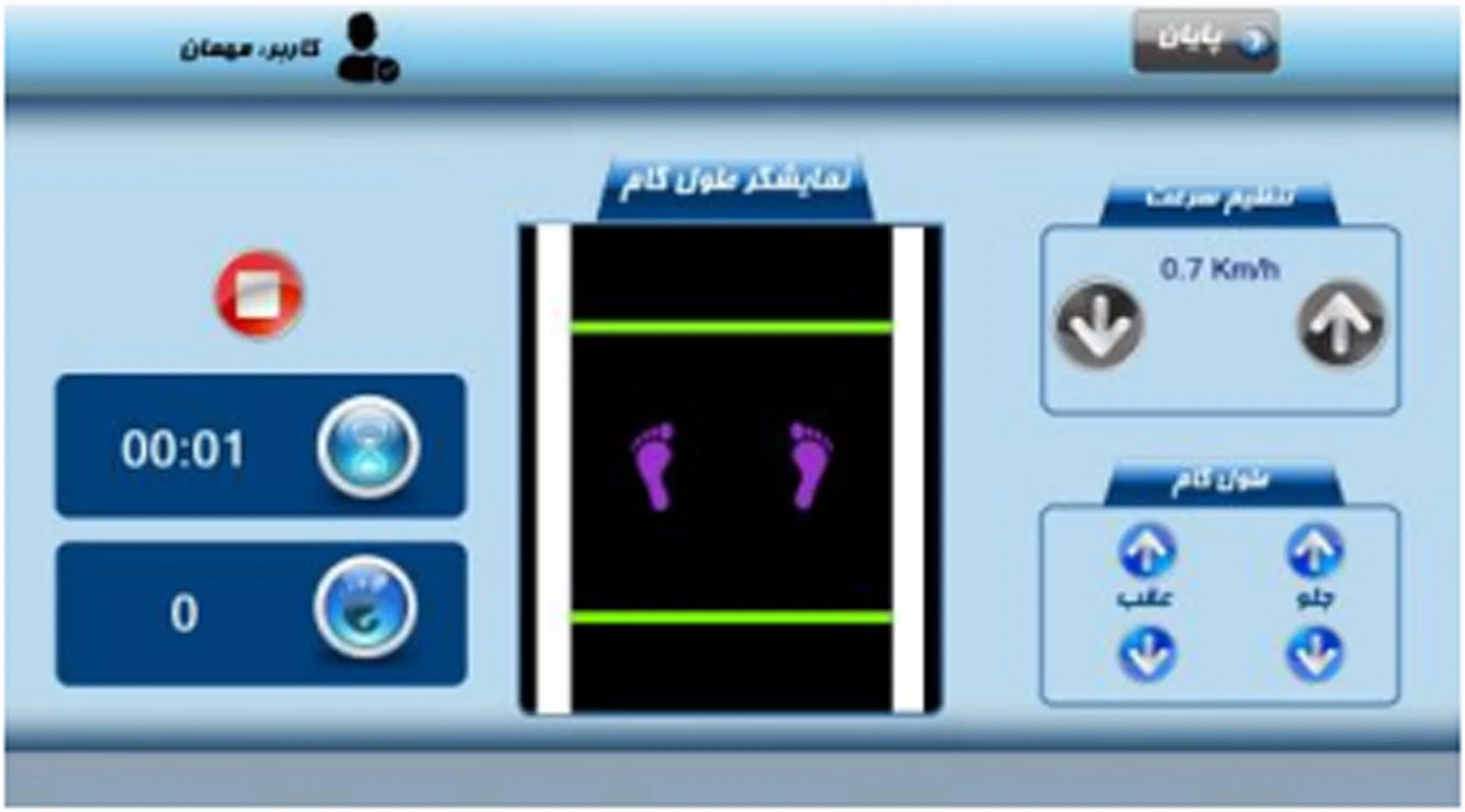
Step lenght.

**Fig. 2:**
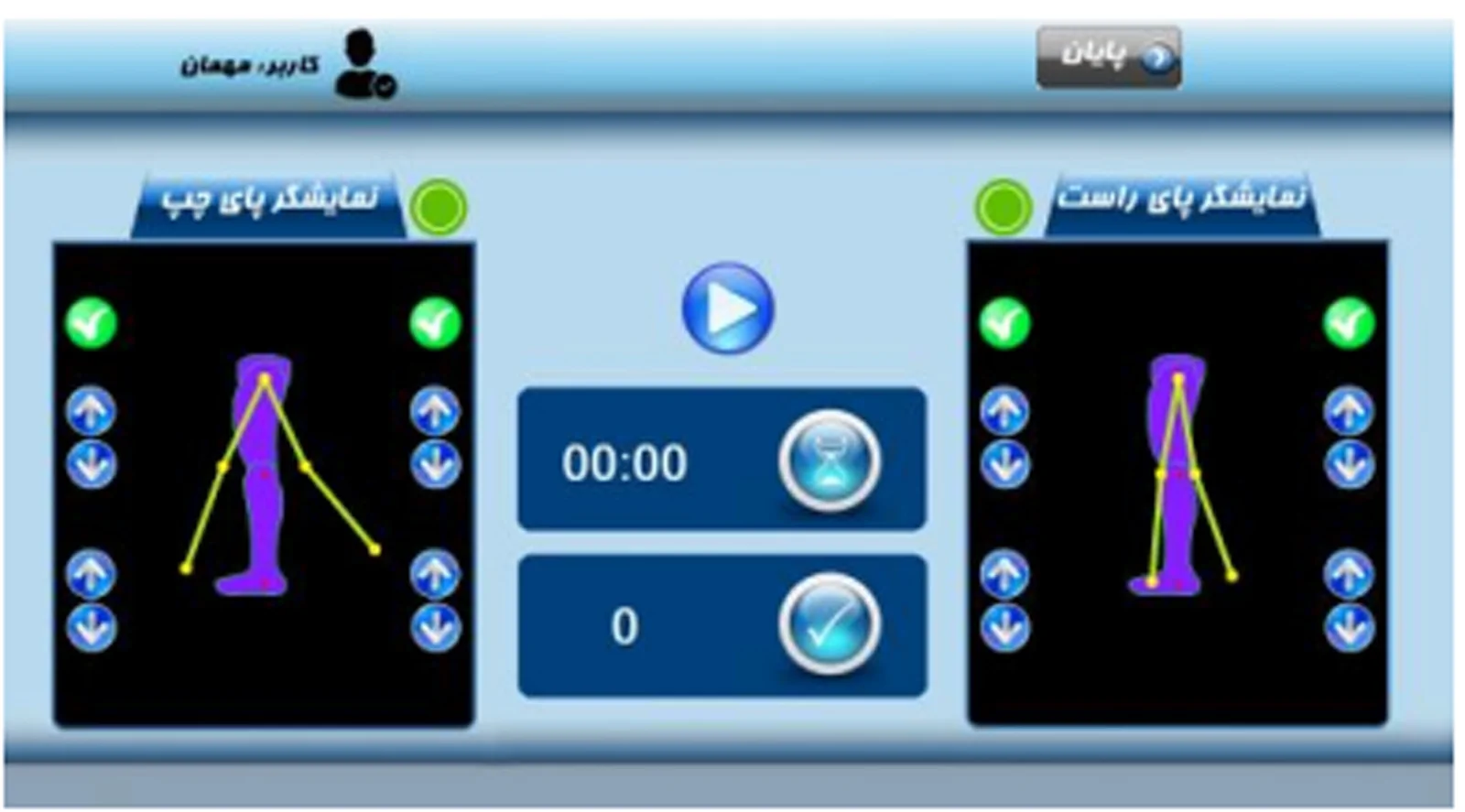
Knee and ankle flexion.

**Fig. 3:**
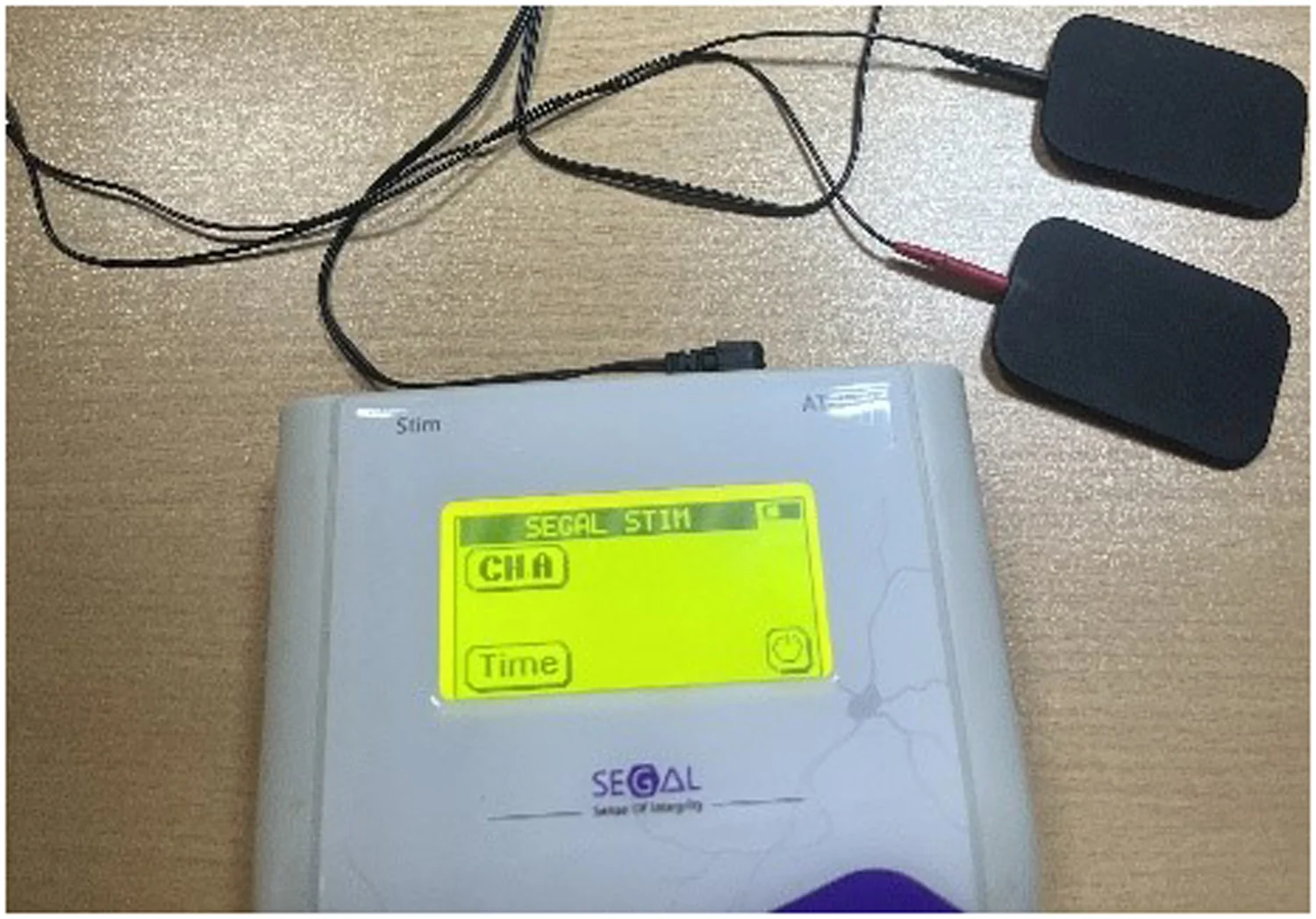
tDCS device.

Participants in the VR group will perform gait training on parallel bars using a virtual reality system. The VR system consisted of a Kinect 2 camera with a motion depth detection range of approximately 5 m, equipped with a moving depth path avatar extraction system. It provided real-time detection and feedback of lower limb joint angles in the sagittal plane, as well as stepping length feedback. VR‑based gait training is delivered in two 10‑minute phases. In the first phase, step length is adjusted using visual cues (green lines) on the VR screen based on the patient’s initial ability, and the patient practices forward and backward parallel walking accordingly (Fig. 1, step lenght). In the second phase, knee and ankle flexion range are adjusted using visual feedback on the screen (Fig. 2, knee and ankle flexion). Training is performed in an open environment, requiring the patient to focus on the VR instructions despite surrounding distractions. tDCS combined with gait training*:* tDCS combined with gait training receive transcranial direct current stimulation simultaneously while walking on the parallel bars for 20 min. The portable and lightweight tDCS device allows stimulation during gait training (Model: Segal Stim AT-12). (Fig. 3, tDCS device). The current intensity is set at 1- 1.5 mA (**as tolerated by the patient),** with the anodal electrode placed over either F3 or F4, depending on the side of the brain lesion, and the cathodal electrode positioned over the contralateral supraorbital area [26]. Typically, stimulation targets the dorsolateral prefrontal cortex (DLPFC), a region associated with the reduction of fear responses [27]. The F3 and F4 locations are identified based on the international 10–20 EEG electrode placement system.

In the first session, participants in both groups performed 1 min of parallel walking with therapist guidance to familiarize them with the intervention procedures. To prevent contamination between groups, Treatments will be scheduled on alternating days (odd and even days).

### Methods of measurement

This study uses the Falls Efficacy Scale International (FES-I) questionnaire to assess fear of falling. The questionnaire comprises 16 items, each scored from 1 (not at all concerned) to 4 (very concerned) [28].

The Mini‑BESTest is a shortened version of the BESTest consisting of 14 items that assess dynamic balance, including anticipatory postural adjustments, reactive postural responses, sensory orientation on level and inclined surfaces, and dynamic gait stability. Each item is scored on a 3‑point ordinal scale (0–2), where 0 indicates inability and 2 indicates normal performance [29].

The Biodex Balance System is used to objectively assess balance. Participants stand barefoot with hip‑width foot placement and try to maintain stability near the center. After familiarization at stability level 8, testing is performed at level 6. Three 20‑second trials with 30‑second rest intervals are completed with eyes open, and the mean of the three trials is used for analysis [30].

The 10‑Meter Walk Test (10MWT) assesses walking speed. Participants walk at their self‑selected comfortable speed along a straight, flat, obstacle‑free 10‑meter walkway, and the time to walk between the 2‑ and 8‑meter markers is recorded to calculate walking speed [31].

Before and after the intervention, an independent physiotherapist blinded to group allocation will assess participants. To minimize learning, fatigue, and bias, assessments will be conducted in random order. All data will then be coded (methods as codes 1 and 2) and analyzed using statistical software.

### Statistics

#### Descriptive statistics

Measures of central tendency (mean) and dispersion (standard deviation, variance, and range) were calculated for quantitative variables.

#### Data analysis

data normality will be assessed with the Shapiro–Wilk test. Depending on distribution, paired t‑tests or Wilcoxon tests will be applied for within‑group comparisons, and 2 × 2 repeated‑measures ANOVA will be used for between‑group analyses. Between-group differences were analyzed using ANCOVA, with time since stroke (post-onset duration) included as a covariate to account for potential differences in recovery status. Statistical analyses will be performed in SPSS v26, with significance set at p < 0.05.

#### Sample size calculation

The primary outcome is fear of falling, measured by the FES‑I. As no MCID^2^ has been established for stroke patients, the MCID was estimated as 1.2 × the MDC (MDC^3^ = 6.69) based on previous studies [28,32].

Based on the standard deviation (SD) reported in the study by Molhemi et al. [9]., and according to the formula, sampsi 40.9 48.9, sd1(8.4) sd2(10.8) pre[1] post[1] r0(0.6) r1(0.6) alpha (0.05) power (0.8), assuming α = 0.05, power = 80%, and a pre–post correlation of 0.6, the calculated sample size was 16 participants per group, accounting for a 15% dropout rate [8,33].

## Protocol validation

Although the validity of this randomized controlled trial protocol is supported by existing scientific evidence and clinical guidelines, Standardized outcome measures with established validity and reliability in the stroke population (FES‑I, Mini‑BESTest and 10MWT) are employed to assess fear of falling, balance, and walking ability, this clinical study is still ongoing, with participant recruitment and intervention currently in progress; therefore, outcome data for empirical validation have not yet been generated.

## Limitations

Although electrode placement followed the international 10–20 EEG system, due to electrode size and proximity to the primary motor cortex (M1), some degree of current spread may have occurred. While the power analysis suggests 16 participants per group, the relatively small sample size may limit the generalizability of the findings; therefore, the results should be interpreted with caution.

## CRediT authorship contribution statement

**Akram Divandari:** Conceptualization, Methodology, Investigation, Software, Data curation, Writing – original draft. **Iraj Abdollahi:** Supervision, Investigation, Conceptualization, Methodology. **Mohammad Hassan Azarsa:** Conceptualization, Methodology, Investigation, Writing – review & editing. **Mohammad Sayyadnasiri:** Visualization, Investigation, Data curation. **Neda Mostafaee:** Conceptualization, Methodology, Investigation, Software, Writing – review & editing.

## Declaration of competing interest

The authors declare that they have no known competing financial interests or personal relationships that could have appeared to influence the work reported in this paper.

## Data Availability

No data was used for the research described in the article.

## Footnotes

1 - Fear Of Falling

2 - Minimal Clinically Important Difference

3 - Minimal Detectable Change
